# Genome-wide characterization of lncRNAs and mRNAs in muscles with differential intramuscular fat contents

**DOI:** 10.3389/fvets.2022.982258

**Published:** 2022-08-08

**Authors:** Yuanlu Sun, Xu Lin, Qian Zhang, Yu Pang, Xiaohan Zhang, Xuelian Zhao, Di Liu, Xiuqin Yang

**Affiliations:** ^1^College of Animal Science and Technology, Northeast Agricultural University, Harbin, China; ^2^Heilongjiang Academy of Agricultural Sciences, Harbin, China

**Keywords:** meat quality, lncRNA, fat deposition, CREB1, adipogenesis

## Abstract

Meat quality is one of the most important traits in pig production. Long non-coding RNAs (lncRNAs) have been involved in diverse biological processes such as muscle development through regulating gene expression. However, studies on lncRNAs lag behind and a comparatively small number of lncRNAs have been identified in pigs. Also, the effects of lncRNAs on meat quality remain to be characterized. Here, we analyzed lncRNAs in longissimus thoracis (LT) and semitendinosus (ST) muscles, being different in meat quality, with RNA-sequencing technology. A total of 500 differentially expressed lncRNAs (DELs) and 2,094 protein-coding genes (DEGs) were identified. Through KEGG analysis on DELs, we first made clear that fat deposition might be the main reason resulting in the differential phenotype of LT and ST, for which cGMP–PKG and VEGF signaling pathways were the most important ones. In total, forty-one key DELs and 50 DEGs involved in the differential fat deposition were then characterized. One of the key genes, cAMP-response element binding protein 1, was selected to confirm its role in porcine adipogenesis with molecular biology methods and found that it promotes the differentiation of porcine preadipocytes, consistent with its higher expression level and intramuscular fat contents in LT than that in ST muscle. Furthermore, through integrated analysis of DELs and DEGs, transcription factors important for differential fat deposition were characterized among which BCL6 has the most target DEGs while MEF2A was targeted by the most DELs. The results provide candidate genes crucial for meat quality, which will contribute to improving meat quality with molecular-breeding strategies.

## Introduction

Meat quality is one of the most important economic traits in pig breeding. With the improvement of living standards, people have paid more and more attention to meat quality. Meat quality parameters determining the visual appearance of meat, such as color, tender, water holding capacity, etc., have become crucial for consumer acceptability, which is an incentive for pig breeders and farmers to improve meat quality. However, meat quality is a comprehensive indicator and most of the traits have low-to-moderate heritability (1, 2). In addition, some items of meat quality are negatively correlated with lean meat percentage and growth traits. Thus, it is difficult to improve meat quality through traditional methods. Molecular-breeding strategies such as marker-assisted selection, gene modification, etc., should be preferred means for which revealing the mechanisms underlying meat quality is the prerequisite.

Numerous efforts have focused on meat quality. It has been shown that *RN, RYR1, PHKG1, IGF2*, and *RAKG3* are major genes controlling meat-quality traits (2–6) and various candidate genes were identified as well (7–9). The development of next-generation sequencing technology made it convenient and efficient to identify candidate genes, especially novel and/or low-abundance transcripts such as long non-coding RNAs (lncRNAs), at the genome-wide level.

Long non-coding RNAs are a class of RNAs with more than 200 nucleotides in length. It was initially identified as mRNA-like transcripts with no protein-coding capability (10). LncRNAs engage in diverse biological processes through regulating gene expression at transcriptional, translational, and post-translational levels (11). LncRNA profiling has been made at a genome-wide level in skeletal muscles and differentially expressed lncRNAs (DELs) were identified with RNA-sequencing (RNA-seq) technology (12–14). But compared to that in humans and mice, studies on lncRNAs lag behind and there are numerous classes of lncRNAs to be identified in pigs. Also, the effects of lncRNAs on meat quality remain to be characterized.

Min pig is a Chinese indigenous breed and excellent in meat quality. Longissimus thoracis (LT) and semitendinosus (ST) exhibit differences in many parameters of meat quality such as tenderness and lightness value (15). Intramuscular fat (IMF) content, correlated positively with meat quality, is also different between LT and ST muscles (16). In addition, LT and ST samples from the same pig can avoid the influence of individual differences and be compared stringently to reveal mechanisms underlying their differential phenotype. Thus LT and ST muscles are good materials for clarification of mechanisms underlying meat quality. To the best of our knowledge, no studies were found aimed at identifying candidate lncRNAs for meat quality in LT and ST muscles. Here, lncRNA profiling was made in LT and ST muscles to characterize DELs with RNA-seq technology, and key lncRNAs and protein-coding genes involved in the formation of fat deposition were found. The results will contribute to further revealing mechanisms underlying meat quality.

## Materials and methods

### Animals, samples, and RNA isolation

Min pigs, a Chinese local pig breeds, were used here and obtained from the Institute of Animal Husbandry, Heilongjiang Academy of Agricultural Sciences, Harbin, China. LT and ST muscles were sampled from three 210-day-old individuals for RNA-seq analysis. Fat tissues were collected from new born piglets for isolating preadipocytes. Total RNA was isolated with TRIzol reagent (Invitrogen, CA, USA), assessed with agarose gel electrophoresis, and quantified with a Nanodrop 2000 (IMPLEN, CA, USA). All the procedures of animal treatment were strictly based on the protocol of the Animal Care Committee of Northeast Agricultural University.

### Library construction and sequencing

Library preparation and RNA-sequencing were performed by Frasergen Inc. (Wuhan, China). In brief, three μg total RNA per sample was used for library construction. rRNA was first removed with Epicentre Ribo-zeroTM rRNA Removal Kit (Epicentre, Madison, WI, USA), and purified with ethanol precipitation. Next, sequencing libraries were constructed with NEBNext^®^ UltraTM Directional RNA Library Prep Kit for Illumina^®^ (NEB, Ipswich, MA, USA) according to the manufacturer's instructions. Then, 150–200 bp long cDNA fragments were selected with the AMPure XP system (Beckman Coulter, Brea, CA, USA). After being treated with USER Enzyme (NEB), PCR was performed with High-Fidelity DNA polymerase, and the products were purified with the AMPure XP system (Beckman Coulter). Afterward, the index-coded samples were clustered on a cBot Cluster Generation System with TrueSeq PE Cluster Kit v3-cBot-Hs (Illumina) based on the manufacturer's instructions. At last, the libraries were sequenced on an Illumina Noveseq platform.

### Raw data processing

Raw reads were first filtered and trimmed using SOAPnuke with –lowQual = 20, –nRate = 0.005, and –qualRate = 0.5 to obtain clean reads. Q20, Q30, and GC contents of the clean reads were calculated by SOAPnuke. Paired-end clean reads were mapped to the reference genome (Sus scrofa 11.1, http://asia.ensembl.org/Sus_scrofa/Info/Index) using HISAT2 (2.1.0) and the coverage of RNA-seq reads was calculated by geneBody_coverage.py script of RSeQC software. Then, mapped reads were assembled using StringTie software in a reference-based approach. The expression level of transcripts was measured with fragments per kilobase of transcript per million fragments (FPKM) mapped, and those with FPKM > 0.1 in at least one sample were used for further analysis.

### LncRNA characterization

Novel lncRNAs were identified by using CNCI (parameters: –m –p 1), CPC2 (default parameter), and PLEK (parameters: –thread 4 –min length 200) softwares simultaneously, and those without coding potential by all of the three tools were considered as candidate lncRNAs. Differentially expressed lncRNAs (DELs) were screened with criteria: the absolute log2(fold change) > 1 and *p*-value < 0.05. Heatmap was plotted by online tools (https://www.bioinformatics.com.cn). To explore the function of lncRNAs, *cis*-target protein-coding genes were predicted within 100,000 bp upstream and downstream of lncRNAs, and they were subjected to Gene Ontology (GO) and Kyoto Encyclopedia of Genes and Genomes (KEGG) pathway analysis.

### mRNA characterization

Differentially expressed mRNAs (DEmRs) were characterized with the same criteria as that for DELs, that is, | log2(fold change) | > 1 and *p*-value < 0.05. Search Tool for the Retrieval of InteractingGenes/Proteins (STRING) was used to reveal the interaction among the differentially expressed protein-coding genes (DEGs), and Cytoscape (Version 3.7.1) was used to visualize the relationship. Based on the minimum required interaction score of 0.7, the network was constructed, of which the core subnetwork was characterized by the radiality analysis. Radiality calculates the correlation between target genes and all nodes, namely, directly and indirectly related nodes, the rank of genes represents the degree of importance of genes in the protein–protein interaction (PPI) network more comprehensively. Parameter as follow: Crad (V)=[∑wϵv ΔG+1-dist(v,w) ]/ *n*-1 (ΔG represents diameter, dist(v,w) represents distance from any node to V, and *n* means total nodes). Transcription factors (TFs) were identified through searching the AnimalTFDB database (http://bioinfo.life.hust.edu.cn/ AnimalTFDB/, version 3.0) with hmmscan program. Target genes of TFs were predicted with Cistrome DB program (http://cistrome.org/db, accessed on 15 April 2022) with a score > 3, and TF-DEG pairs with a Pearson correlation coefficient in expression level > 0.8 were selected for further analysis.

### Real-time quantitative PCR

Reverse transcription (RT) was performed with the PrimeScript^TM^ RT Reagent Kit (TaKaRa, Dalian, China) to synthesize cDNA according to the manufacturer's instructions. Real-time quantitative PCR (qPCR) was conducted with ChamQ Universal SYBR qPCR Master Mix (Vazyme, Nanjing, China) according to the manufacturer's instructions, each with three replicates. β-actin gene was used as a reference and 2–ΔΔCt (17) method was used to calculate the relative expression level of target genes. Primers used in qPCR was given in Supplementary Table S1.

### Preadipocyte culture, differentiation, and oil red O staining

Primary preadipocytes culture and differentiation induction were described previously (18). Briefly, subcutaneous fat tissues were sampled from the newborn Min pigs and digested with 0.1% type I collagenase (Invitrogen), and then filtered through 400-mesh filters. The cells obtained were cultured in DMEM/F12 medium (Dulbecco's modified Eagle's medium/Nutrient Mixture F-12) containing 10% fetal bovine serum (FBS) and 1% penicillin–streptomycin. The medium was changed every 2 days.

For differentiation induction, cells were first cultured in DMEM/F12 medium supplemented with 10% FBS, 0.5 mmol/L 3-isobutyl−1-methylxanthine, 1 μmol/L dexamethasone and 5 μg/ml insulin for 2 days and then transferred into DMEM/F12 medium containing 10% FBS and 5 μg/ml insulin to maintain the differentiation.

The adipocytes were stained with an Oil Red O kit (Leagene, Beijing, China), and then viewed under a light microscope and photographed (Carl Zeiss AG, Jena, Germany). Cellular Oil Red O was then isolated with isopropanol and quantified with optical absorbance at 510 nm.

### Transiently transfection

The coding sequence of the porcine cAMP-response element-binding protein 1 (CREB1) gene was amplified and subcloned into the pCMV-HA vector at enzyme sites EcoRI and XhoI to construct overexpression plasmids. SiRNA sequences were designed and synthesized by General Biol (Anhui, China) to knock down the expression of *CREB1* in preadipocytes. The optimal siRNA sequence, selected through preliminary experiments, was given in Supplementary Table S1. In total, 30 nmol/μl of siRNA or 0.5 μg of overexpression plasmids were transiently transfected into preadipocytes with Lipofectamine 2000 reagent (Invitrogen) according to the manufacturer's instructions. At 24 h after transfection, the cells were induced to differentiation. The cell culture medium was changed every 2 days and at 8 days post-induction, cells were stained with Oil Red O or collected to measure the expression of the adipogenic marker gene, peroxisome proliferator-activated receptor (PPAR)γ and CCAAT/enhancer-binding protein (C/EBP)α, with the qPCR method.

### Statistical analysis

All the experiments were repeated at least three times independently, each with triplicate. Data were given as mean ± standard error. SPSS 19.0 software was used to analyze the data. The Student's *t*-test was performed to compare the difference between the two groups. A *p* < 0.05 was considered to be statistically significant. Significant difference was indicated with ^*^ (*p* < 0.05) and ^**^ (*p* < 0.01).

## Results

### Overview of lncRNA sequencing

After filtering out redundant and low quality reads, 65.36 and 68.66 million clean read pairs were obtained in LT and ST muscles on average, respectively, comprising at least 98% of Quality 20 (Q20) reads and 94.4% of Q30 reads. In each sample more than 94.9% clean reads were mapped to reference genome (Sus scrofa 11.1) (Supplementary Table S2-1).

**Figure 1:**
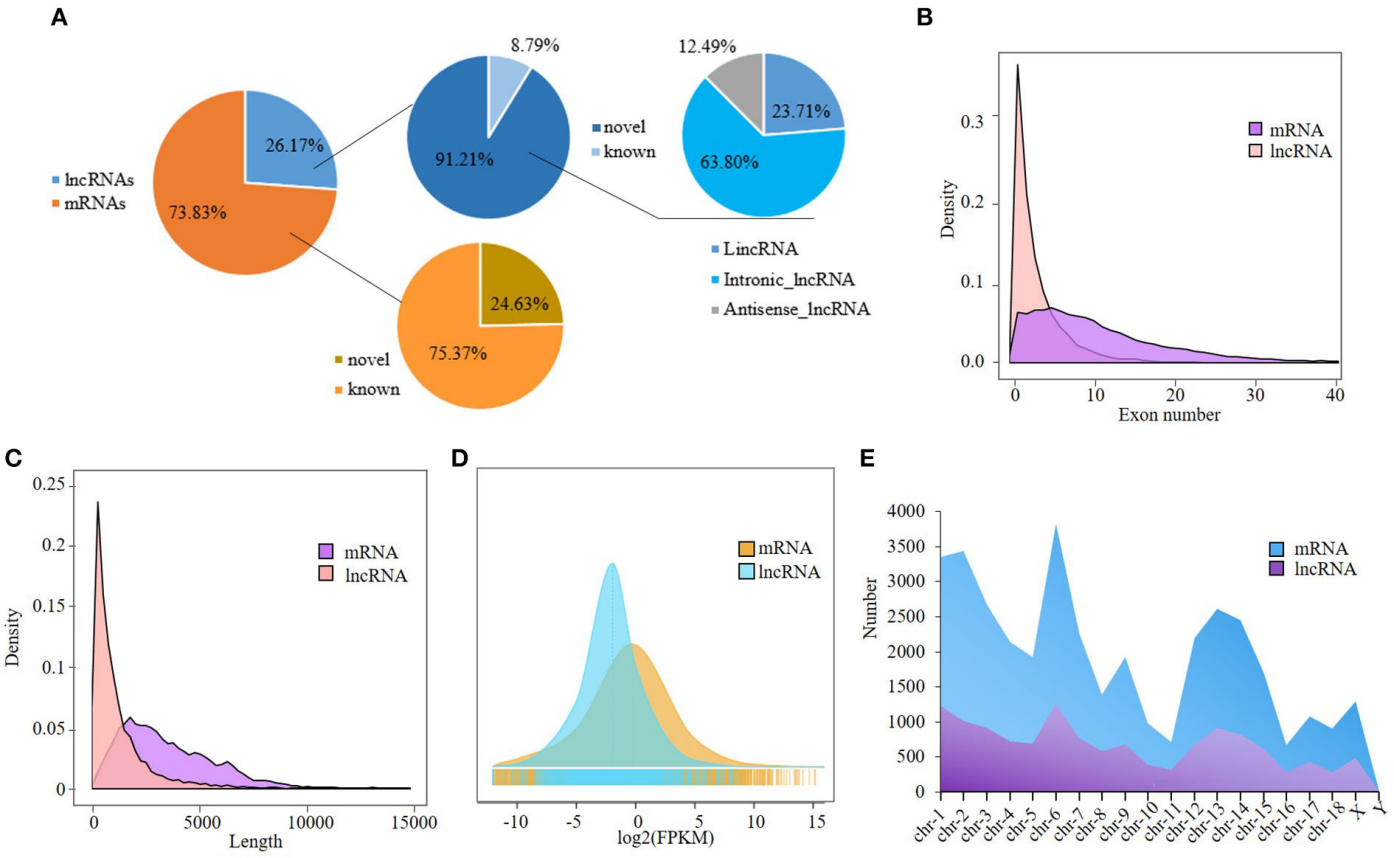
Analysis of RNA-seq data. **(A)** Classification of lncRNAs and mRNAs; **(B,C)** Transcript length and exon number distribution of the lncRNAs and mRNAs; **(D)** Distribution of the lncRNAs and mRNAs in chromosomes; **(E)** Expression level distribution of the lncRNAs and mRNAs.

A total of 13,635 lncRNAs and 38,468 mRNAs were obtained. Among them, 12,437 lncRNAs were novel as identified by CNCI, CPC, and PLEK programs, and 9473 mRNAs were novel, accounting for 91.21 and 24.63% of total lncRNAs and mRNAs, respectively (Supplementary Table S2-2). The majority of the novel lncRNAs were intronic, and antisense lncRNAs were the least (Figure 1A). The novel lncRNAs ranged from 201 to 31,717 bp in length with an average of 1,168 bp, and were composed of 2–66 exons with an average number of 4.5 (Supplementary Table S2-3). In general, lncRNAs are shorter than mRNAs in length and composed of fewer exons. The distributions of exon number and length of lncRNAs and mRNAs were shown in Figures 1B,C. The expression level of lncRNAs is lower than that of the mRNAs (Figure 1D). Although the abundance of unique lncRNAs is much lower than that of mRNAs in chromosomes, they have similar distribution: both the lncRNAs and mRNAs were mainly distributed on chromosomes 1 and 6 (Figure 1E).

### Differentially expressed lncRNAs and their functions

**Figure 2:**
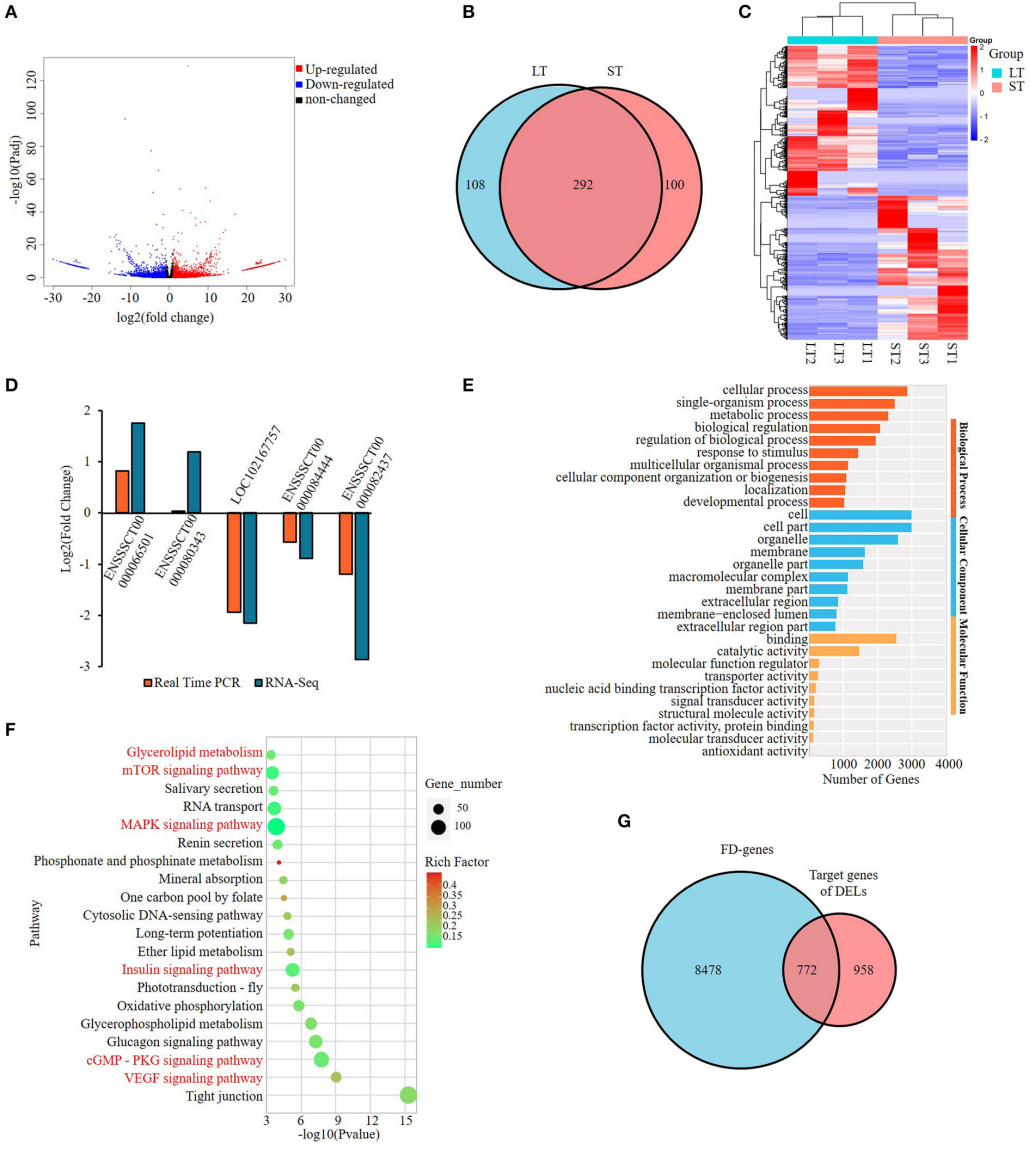
Screening and enrichment analysis of the differentially expressed lncRNAs (DELs) in semitendinosus compared with longissimus thoracis. **(A)** Volcano plot of DELs; **(B)** Venn diagram of DELs; **(C)** Hierarchical clustering heatmap of the top DELs; **(D)** Real-time PCR validation of DELs; **(E)** Top 10 GO terms enriched by *cis*-target genes of DELs in each category; **(F)** Top 20 KEGG pathways significantly enriched by the *cis*-target genes of DELs. Fat-related pathways were indicated with red; **(G)** Venn diagram of *cis*-target genes of DELs and fat deposition genes.

To identify lncRNAs involved in the differential phenotype of two muscles, RNA-seq was performed and a total of 500 DELs were obtained with 245 upregulated and 255 downregulated in ST compared with LT muscle (Supplementary Table S3-1). Among the DELs, 108 were specific to ST tissue and 100 were specific to LT tissue (Figures 2A,B). Heatmap of top DELs were shown in Figure 2C. The differential expression of five DELs was validated by real-time quantitative PCR (qPCR) and consistent results were obtained except for one lncRNA, ENSSSCT00000080343. It has a tendency to increase in ST compared with LT, but the difference was not as obvious as that in RNA-seq (Figure 2D). The results indicate that RNA-seq data was reliable.

Gene ontology and KEGG analyses were applied on the target genes of DELs. A total of 2,401 GO terms involved in categories of cellular component (CC), biological process (BP), and molecular function (MF) were enriched (Supplementary Table S3-2). Cell and cell part were the GO terms enriched with the most genes among all three categories, while in BP category metabolic process is the most highly enriched GO terms with over 2,000 genes (Figure 2E). The KEGG analysis showed that DELs were mainly enriched in fat-related pathways significantly (*p* < 0.05) (Supplementary Table S3-3). Of the top 20 pathways, six were involved in adipogenesis, namely, VEGF signaling pathway, cGMP-PKG signaling pathway, Insulin signaling pathway, MAPK signaling pathway, mTOR signaling pathway, and Glycerolipid metabolism, among which VEGF and cGMP–PKG ranked top 2 and 3, respectively (Figure 2F).

To further identify the role of DELs, we downloaded 9,250 fat deposition (FD) genes from GeneCards database (https://www.genecards.org/, accessed on 15 Apr 2022) and found that 772 target genes of DELs were included in the FD gene list, representing 44.6% of all target genes of DELs (Figure 2G; Supplementary Table S3-4). Of the 500 DELs identified, 71.6% (358) have target genes belonging to the list and were named FD–DELs here (Supplementary Table S3-5). In addition, 59.5% of target genes involved in the top 20 significantly enriched pathways were FD genes (Supplementary Table S3-6). These results suggest that fat deposition might be the main reason resulting in the differential phenotype of two muscles. Thereafter, we will focus on FD-genes to reveal the difference between the two muscles in the following analysis.

### Analysis of differentially expressed protein-coding genes and their functions

**Figure 3:**
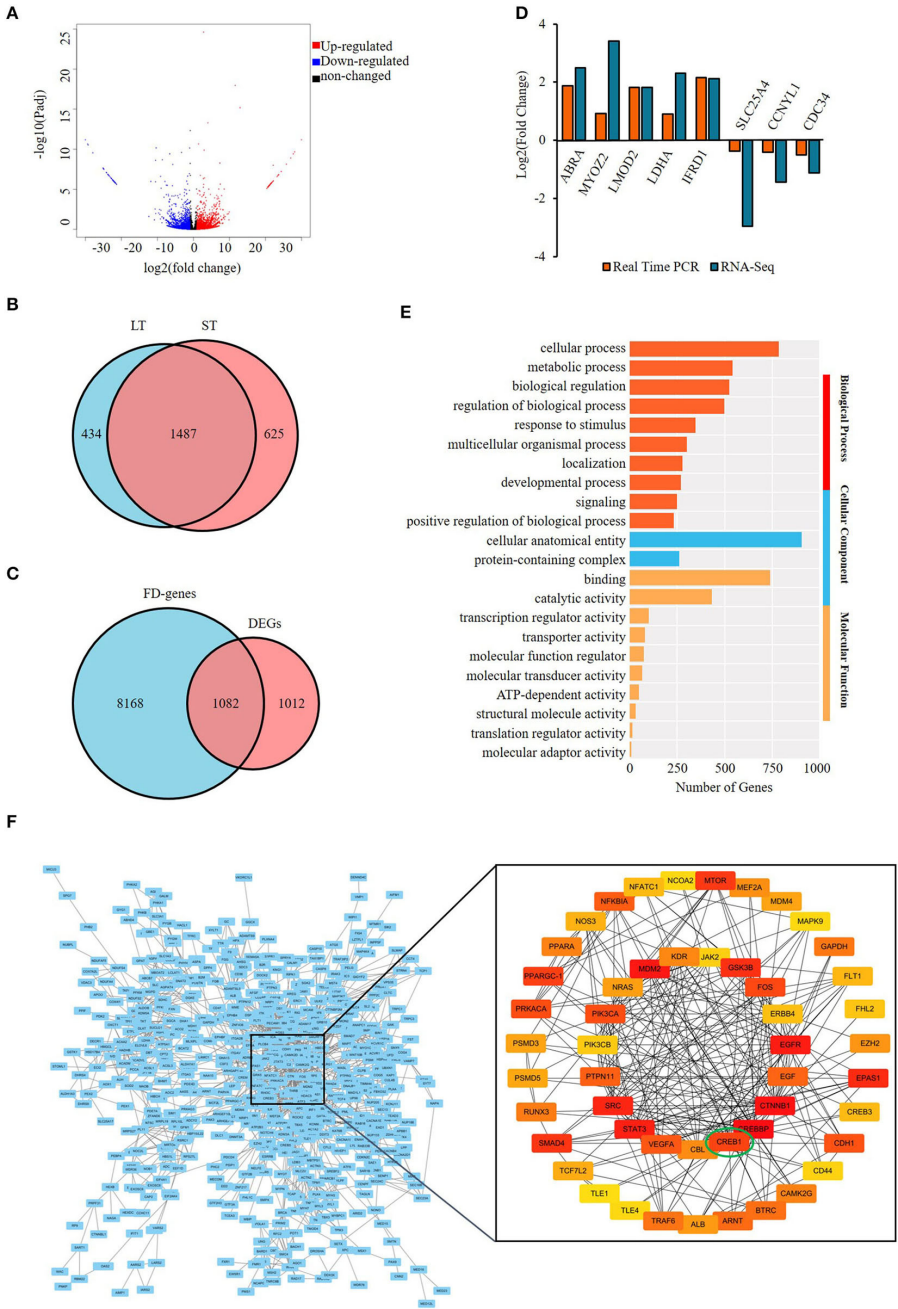
Screening and enrichment analysis of the differentially expressed genes (DEGs) in semitendinosus compared with longissimus thoracis. **(A)** Volcano plot of DEGs; **(B)** Venn diagram of differentially expressed mRNAs; **(C)** Venn diagram of DEGs and fat deposition genes; **(D)** real-time PCR validation of DE mRNA; **(E)** Top 10 GO terms enriched by FD–DEGs in each category.; **(F)** Protein–protein analysis of FD–DEGs. The minimum interaction score was set as 0.7. The color of the circle represents the degree of importance.

A total of 2,094 differentially expressed protein-coding genes (DEGs), covering 2,546 DEmRs were identified. Among the DEmRs, 1,495 were upregulated and 1,051 downregulated in the ST compared with LT (Figure 3A) (Supplementary Table S4-1); 625 and 434 were specifically expressed in ST and LT, respectively (Figure 3B). The intersection of DEGs and FD genes showed that 1,082 were FD–DEGs, accounting for 51.2% of total DEGs (Figure 3C; Supplementary Table S4-2). The differential expression of eight DEGs was validated with qPCR, and consistent results were obtained (Figure 3D).

The FD–DEGs were enriched in various GO terms involved in BP, MF, and CC (Supplementary Table S4-3). In the BP category, cellular process is the most highly enriched terms with 786 genes (Figure 3E). To explore the interaction among the FD–DEGs identified, PPI analysis was performed and genes with a score > 0.7 were visualized with cytoscape (Version 3.7.1) (Figure 3F). The subnetwork constructed consists of 50 nodes and 230 edges. The number of edges in each node is ranged from 2 to 30. Epidermis growth factor (EGF) receptor (EGFR), signal transducer, and activator of transcription 3 (STAT3), cAMP-response element binding protein 1 (*CREB1*)-binding protein (CREBBP) and catenin beta 1 have the most edges. *CREB1* has 12 edges and is ranked 12 among all the genes. These 50 genes, namely, PPARα, PPARγ coactivator-1 (PPARGC-1), EGF, vascular endothelial growth factor A, and *CREB1* should be the key protein-coding genes engaged in the differential deposition of fat between two muscles (Supplementary Table S4-4).

### Effects of CREB1 on porcine adipogenesis

**Figure 4:**
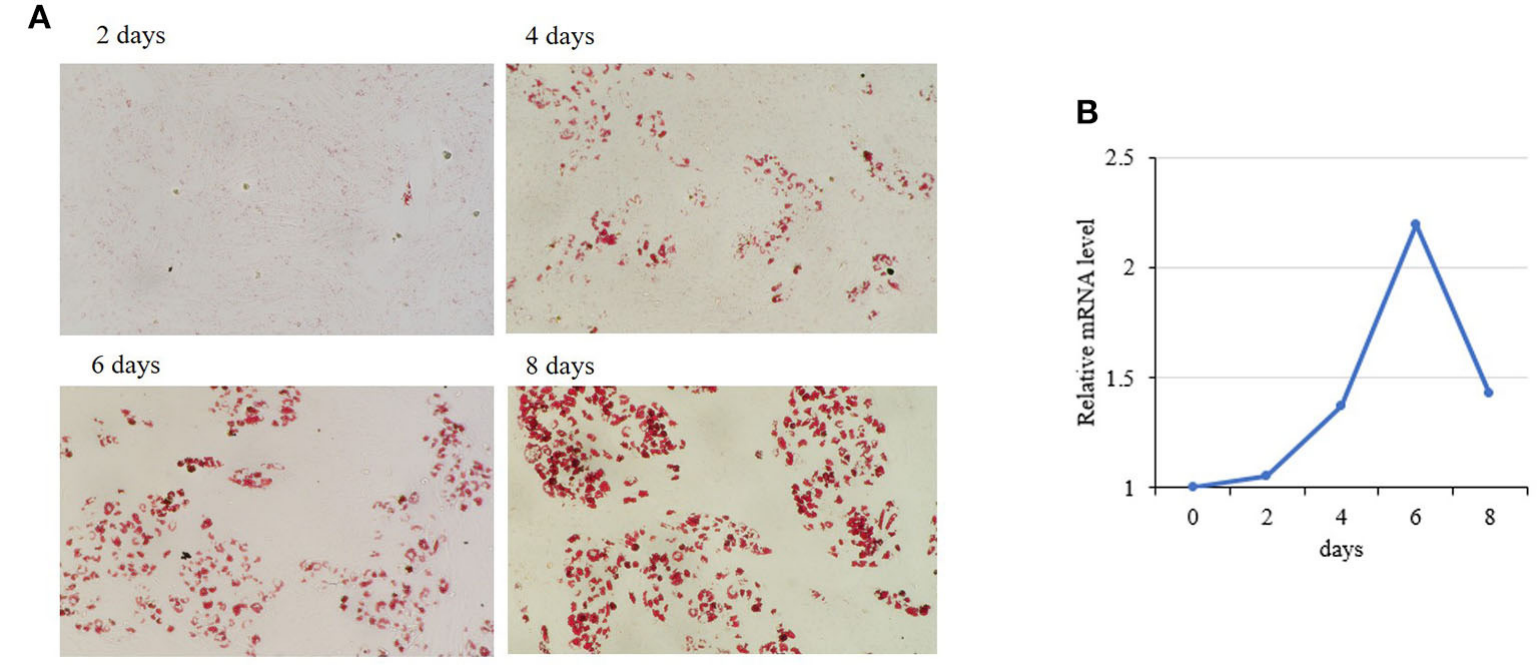
Expression of porcine *CREB1* during differentiation of preadipocytes. **(A)** Oil Red O staining of preadipocytes during differentiation induction; **(B)** relative mRNA level of *CREB1* during differentiation of preadipocytes.

**Figure 5:**
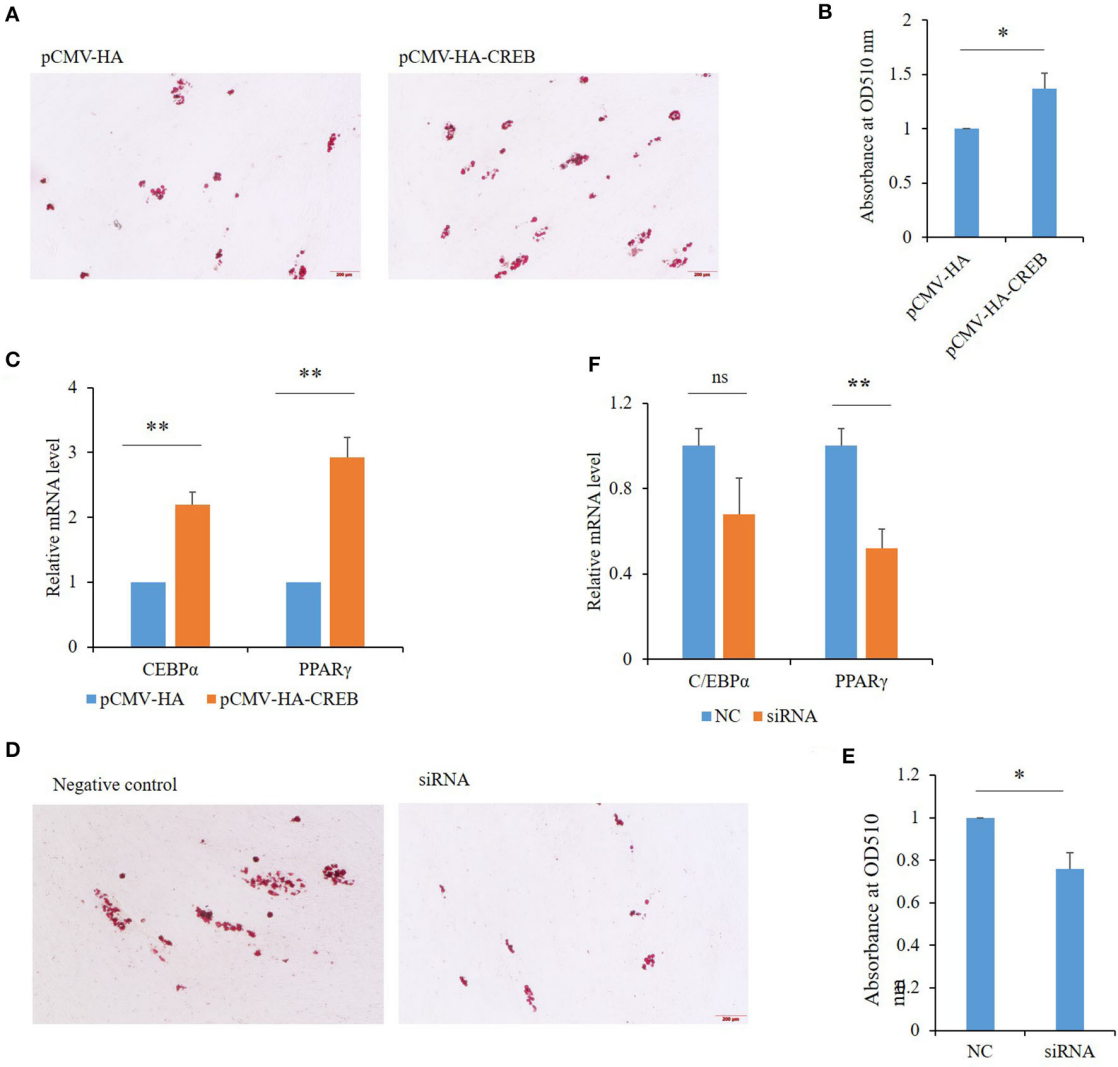
Effects of *CREB1* on porcine preadipocyte differentiation. **(A)** Oil Red O staining of preadipocytes overexpressing *CREB1* gene at 8 days post-induction; **(B)** quantitative analysis of triglyceride contents in preadipocytes overexpressing *CREB1* gene with optical absorbance; **(C)** expression of C/EBPα and PPARγ in preadipocytes overexpressing *CREB1* gene; **(D)** Oil Red O staining of preadipocytes knocked down for *CREB1* gene at 8 days post-induction; **(E)** quantitative analysis of triglyceride contents in preadipocytes knocked down for *CREB1* gene with optical absorbance; **(F)** expression of C/EBPα and PPARγ in preadipocytes knocked down for *CREB1* gene. *p < 0.05, **p < 0.01.

Among the 50 key DEGs, *CREB1* was only expressed in LT muscle which has more intramuscular fat (IMF) content than that of ST. To confirm the role of these key DEGs in the differential fat deposition between two muscles, we analyzed *CREB1* in primary cultured porcine preadipocytes. Oil Red O staining showed that the preadipocytes were induced to differentiation successfully (Figure 4A). During the preadipocyte differentiation, the expression of *CREB1* increased gradually with the highest level at 6-days post-induction (Figure 4B). Overexpression and knockdown of *CREB1* were used to explore the functions and transfection efficiency was shown in Supplementary Figure S1. Overexpression of *CREB1* promotes the differentiation of preadipocytes as revealed by Oil Red O staining at 8 days post-induction (Figure 5A), which was confirmed with quantification assay (Figure 5B). Furthermore, the relative mRNA level of the adipogenic marker, PPARγ, and C/EBPα, increased significantly (*p* < 0.01) (Figure 5C). Consistently, knockdown of *CREB1* by siRNA inhibits the differentiation of preadipocytes as revealed by both Oil Red O staining and quantification assay (Figures 5D,E), and the relative mRNA level of PPARγ was decreased significantly (*p* < 0.01) (Figure 5F). Thus, *CREB1* promotes the differentiation of porcine preadipocytes, that is, promotes adipogenesis, which is consistent with its higher expression level and fat contents in LT than that in ST muscle.

### Integrated analysis of DELs and DEGs

#### Characterization of key lncRNAs

**Figure 6:**
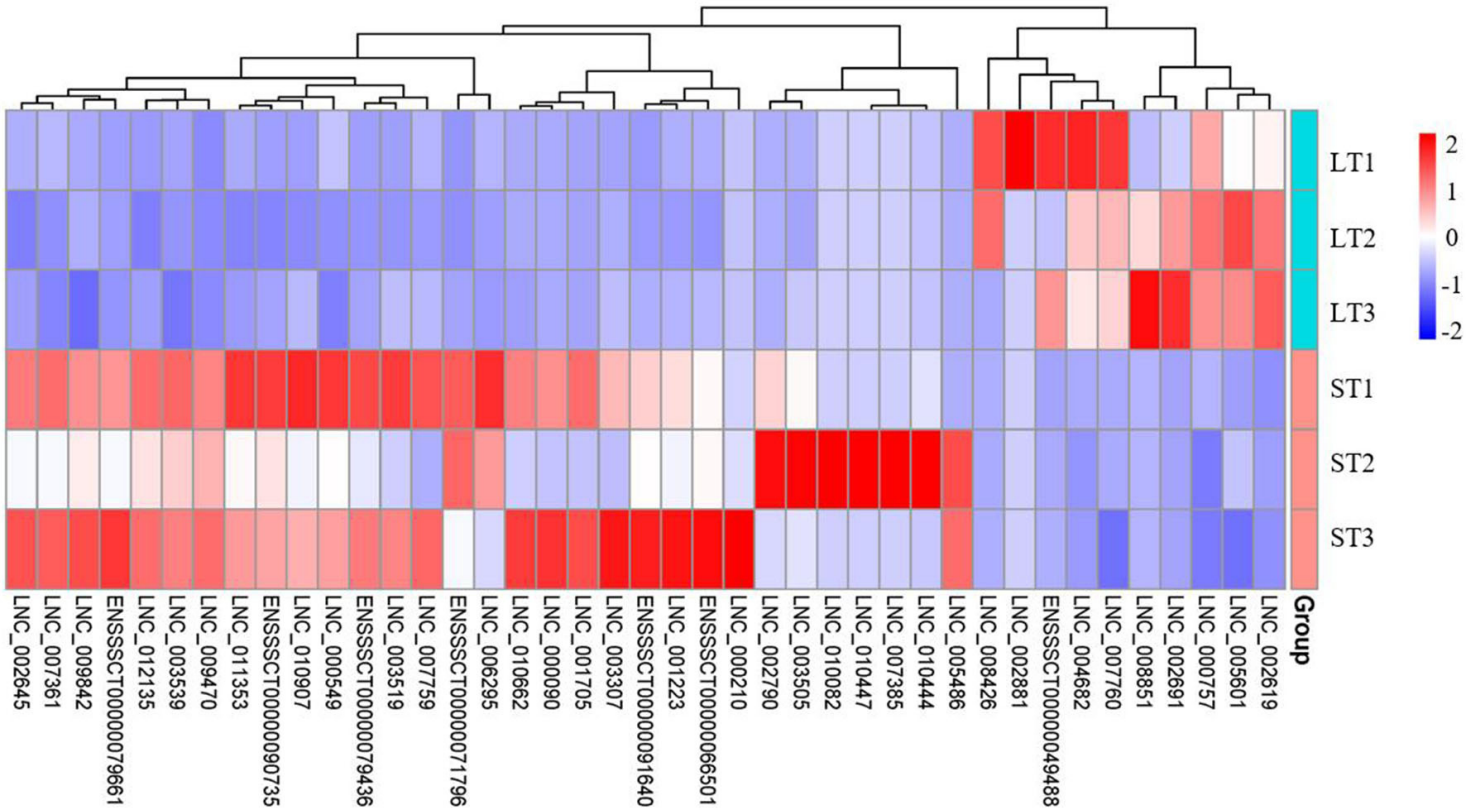
Hierarchical clustering heatmap of key lncRNAs.

Through integrated analysis of FD–DELs and FD–DEGs, we found that 142 FD–DELs have *cis*-target genes covered by FD–DEGs. Coexpression analysis showed that 41 of 142 FD–DELs have a correlation coefficient > 0.9 with target genes. These 41 FD–DELs were expressed stably among samples, and thereafter, can be used as key lncRNAs in future studies revealing the mechanisms underlying the differential fat contents between two muscles (Figure 6).

#### Transcription factor characterization

**Figure 7:**
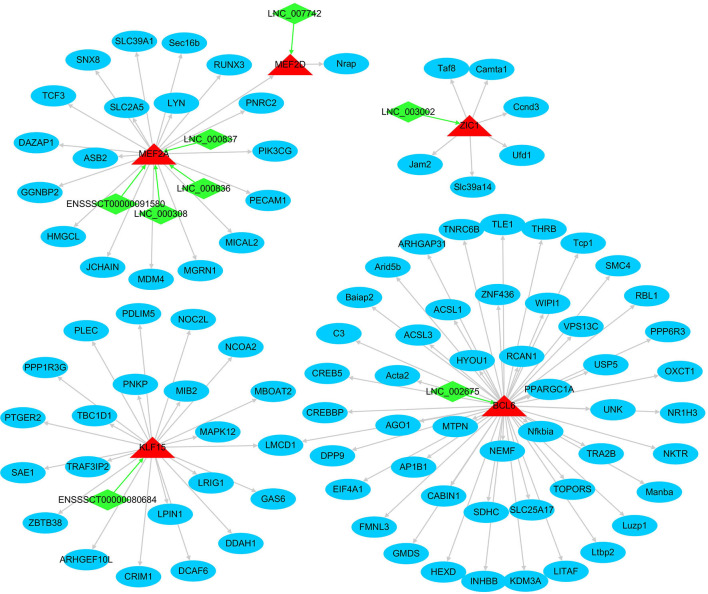
The network formed by fat deposition (FD)-differentially expressed transcription factors, FD-differentially expressed lncRNAs, and FD-differentially expressed genes. Arrows indicate the target direction. The green quadrilateral represents lncRNA, the red triangle represents TF, and the blue circle represents mRNA.

In total, 265 out of the FD–DEGs were characterized as TFs, that is, FD–DETFs, through searching the AnimalTFDB database with hmmscan program (Supplementary Table S5-1). Among the FD–DETFs, 26 were *cis*-targets of FD–DELs, corresponding to 33 FD–DELs (Supplementary Table S5-2). In addition, target genes of the 26 FD–DETFs were characterized by Cistrome DB program with a score of > 3 and DETF-DEG pairs with a Pearson correlation coefficient of > 0.8 were selected (Supplementary Table S5-3). To reveal the relationship between these FD–DETFs, FD–DELs, and FD–DEGs, a network was constructed. A total of five FD–DETFs, eight FD–DELs and 95 FD–DEGs were included in the network in which BCL6 has the most target FD–DEGs, while MEF2A was targeted by the most FD–DELs (Figure 7).

## Discussion

Intramuscular fat deposition is crucial for improving meat quality as its content is positively correlated with tenderness, juiciness, flavor, etc. (19). Meat marbling, a continuing demand for livestock production, is determined by IMF content. There is an obvious difference between LT and ST muscles in IMF content and lipid deposition: LT has higher IMF content and earlier deposition of lipid than ST in pigs (16). Here, through genome-wide analysis of lncRNAs involved in the differential phenotype of LT and ST, we first made clear that fat deposition in them, that is, IMF, might be the main reason leading to the difference, and that cGMP–PKG and VEGF signaling pathways were the most important pathways regulating the difference. The key DELs and DEGs related to fat deposition were then identified, and the involvement of these key genes in adipogenesis in pigs was validated with molecular biological methods in which *CREB1* was taken as an example and consistent results were obtained. In addition, TFs important for differential fat deposition were characterized. All in all, we provide candidate genes for further clarifying the mechanisms underlying meat quality.

Owing to its importance in improving meat quality, many studies have focused on revealing the mechanisms underlying IMF content, and some lncRNAs have been implicated in IMF content in recent years, namely, LncIMF4 (20), IMFNCR (21), IRLnc (22), IMFlnc1 (23), etc. Efforts were also made to characterize lncRNAs involved in IMF deposition at the genome-wide level in pigs (24–26). However, IMF is a highly complicated and metabolically active trait in which genetic factors involved are distinctive and diverse. There are often differences in the genetic cause of IMF accumulation, and the association between genetic markers and IMF content is not always consistent between breeds. Thereafter, it is necessary to identify lncRNAs related to the IMF content in pig breeds such as Min that have not been studied. To the best of our knowledge, no studies focused on revealing lncRNAs involved in differential IMF contents between LT and ST muscles yet. We identified 12,437 novel lncRNAs in the skeletal muscles of Min pigs further indicating the heterogeneous and diverse of lncRNAs. LncRNAs in Min pigs share similarities in structure and classification with their counterparts in mammals (27–29). The number of lncRNAs identified here is somewhat higher than that in other pig breeds such as Duroc, Landrace, and Guizhou miniature pigs (30–33), which might be caused by the rich genetic resource in Min pigs, and also by the different screening criteria used for lncRNA characterization.

A total of 500 DELs were identified between LT and ST muscles in Min pigs. Through integrated analysis of DELs and DEGs, 41 key lncRNAs related to differential IMF content between LT and ST muscles were characterized. Only seven of the key lncRNAs were known, indicating enormous genetic information involved in IMF deposition remains to be identified. These key lncRNAs will be the emphasis in clarifying the differential IMF content between LT and ST muscles in the future study. In addition, 50 key DEGs engaged in differential fat deposition between the two muscles were characterized including widely known fat-related genes such as PPARα (34), PPARGC-1 (35), mTOR (36), EGF and its receptor, EGFR (37).

*CREB1* and the binding protein, CREBBP, genes were also identified as key factors influencing the fat deposition in LT and ST muscles. *CREB1*, also named CREB, has been involved in adipogenesis in mice (38–40). Expression of constitutively active CREB alone in 3T3-L1 cells was sufficient to induce adipogenesis, while ectopic expression of a dominant-negative CREB protein blocked adipogenesis effectively in cells treated with differentiation-inducing agents (38). Depletion of *CREB1* inhibits the adipogenic conversion of 3T3-L1 cells overexpressing C/EBPα, C/EBPβ, or PPARγ2 (39). *CREB1* drives the expression of several adipocyte-specific genes such as fatty acid binding protein and fatty acid synthetase through binding to the putative cAMP response elements in the promoter (38, 41). However, all the aforementioned studies were performed in cell lines, 3T3-L1, and little was known about pig *CREB1*. Here, in primary-cultured porcine preadipocytes, we showed that porcine *CREB1* promotes adipogenesis. The results not only confirmed the reliability of data characterized by bioinformatic approaches here but contribute to further revealing the role of CREB1 in adipogenesis.

Adipogenesis is a highly orchestrated process of cell differentiation from preadipocytes to mature adipocytes in which TFs are the best-understood regulators (42). It is an elegant progression controlled by the TF cascade which is followed by the expression of adipocyte genes. The sequential activation of TFs is the prerequisite for adipogenesis. To date, many TFs such as PPARγ, C/EBP, and Krüppel-like factors, etc., have been found to play important roles in preadipocyte differentiation. These TFs come from a large variety of families and have many different DNA-binding domains, which provide the basis for transcriptionally activating the expression of a large number of adipocyte genes. Although substantial ongoing progress has been made in our understanding of adipogenesis, more regulators of adipocyte development including TFs remain to be identified (42). Here, through integrated analysis of DELs and DEGs, we characterized important TFs, and their interactive genes and lncRNAs involved in the differential fat deposition between LT and ST muscles. It is worth revealing their role in IMF deposition with molecular biology approaches.

## Data availability statement

The datasets presented in this study are deposited in the NCBI GEO repository, accession number: GSE207449.

## Ethics statement

The animal study was reviewed and approved by Laboratory Animal Welfare and Ethics Committee of Northeast Agricultural University.

## Author contributions

XY and DL: funding acquisition and writing–reviewing. XY: conceptualization. YS, XL, QZ, XZhan, and XZhao: investigation. YP and XL: data analysis. XY and YS: writing. All authors contributed to the article and approved the submitted version.

## Funding

This research was funded by the National Natural Science Foundation of China (32172696) and the China Postdoctoral Science Foundation (2020M670876). Heilongjiang operation expenses for scientific research (CZKYF2020A004).

## Conflict of interest

The authors declare that the research was conducted in the absence of any commercial or financial relationships that could be construed as a potential conflict of interest.

## Publisher's note

All claims expressed in this article are solely those of the authors and do not necessarily represent those of their affiliated organizations, or those of the publisher, the editors and the reviewers. Any product that may be evaluated in this article, or claim that may be made by its manufacturer, is not guaranteed or endorsed by the publisher.

## Supplementary material

Supplementary Figure S1 Efficiency of overexpressing or knockdown of *CREB1* in preadipocytes.

Supplementary Table S1 Primers used for real-time quantitative PCR.

Supplementary Table S2 Overview of RNA-seq data.

Supplementary Table S2-1 Quality of RNA-seq results.

Supplementary Table S2-2 Novel lncRNAs identified.

Supplementary Table S3 Analysis of differentially expressed lncRNAs.

Supplementary Table S3-1 Differentially expressed lncRNAs identified.

Supplementary Table S3-2 GO terms enrichment by differentially expressed lncRNAs.

Supplementary Table S3-3 KEGG pathways enriched by differentially expressed lncRNAs.

Supplementary Table S3-4 Target genes of differentially expressed lncRNA.

Supplementary Table S3-5 Differentially expressed lncRNAs involved in fat deposition.

Supplementary Table S3-6 Target genes involved in top 20 KEGG pathways.

Supplementary Table S4 Analysis of differentially expressed mRNAs.

Supplementary Table S4-1 Differentially expressed mRNAs identified.

Supplementary Table S4-2 Differentially expressed genes involved in fat deposition.

Supplementary Table S4-3 GO terms enriched by FD-DEGs.

Supplementary Table S4-4 Key protein-coding genes identified.

Supplementary Table S5 Characterization of transcription factors.

Supplementary Table S5-1 Differentially expressed transcription factors involved in fat deposition.

Supplementary Table S5-2 FD-DETFs belonging to cis-targets of FD-DELs.

Supplementary Table S5-3 Important DEGs targeted by FD-DETFs.
