# Supplementary figures and images for: Genome-wide characterization of lncRNAs and mRNAs in muscles with differential intramuscular fat contents
Source: Front Vet Sci. 2022 Aug 8;9:982258. doi: 10.3389/fvets.2022.982258 (PMC9393339; doi:10.3389/fvets.2022.982258)

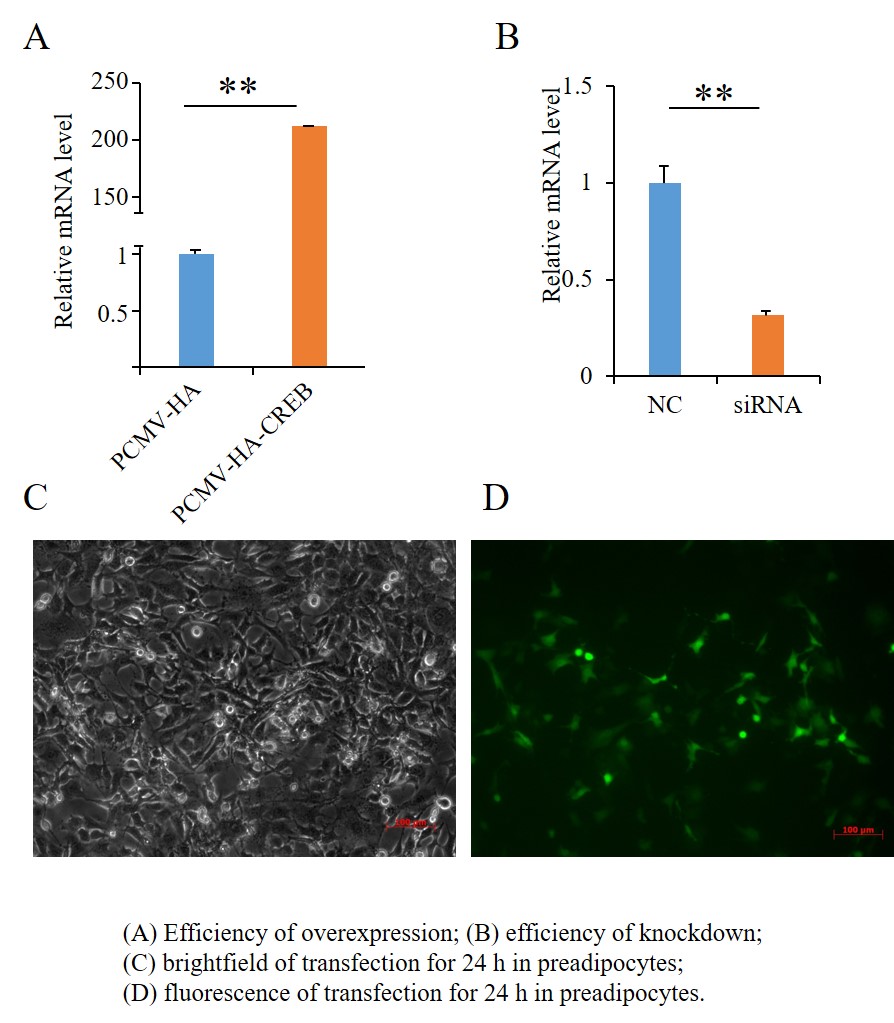

Supplement: Supplementary Figure S1 — Efficiency of overexpressing or knockdown of CREB1 in preadipocytes. [file Data_Sheet_1.ZIP › Supplementary Materials/Supplementary Figure 1.jpg]
